# ENU-mutagenesis mice with a non-synonymous mutation in *Grin1* exhibit abnormal anxiety-like behaviors, impaired fear memory, and decreased acoustic startle response

**DOI:** 10.1186/1756-0500-6-203

**Published:** 2013-05-21

**Authors:** Juzoh Umemori, Keizo Takao, Hisatsugu Koshimizu, Satoko Hattori, Tamio Furuse, Shigeharu Wakana, Tsuyoshi Miyakawa

**Affiliations:** 1 Division of Systems Medical Science, Institute for Comprehensive Medical Science, Fujita Health University, 1-98 Dengakugakubo Kutsukake-cho, Toyoake 470-1192, Japan; 2 Japan Science and Technology Agency (JST), Core Research for Evolutional Science and Technology (CREST), Kawaguchi, Japan; 3 Section of Behavior Analysis, Center for Genetic Analysis of Behavior, National Institute for Physiological Sciences, Okazaki, Japan; 4 Genetic Engineering and Functional Genomics Group, Frontier Technology Center, Kyoto University Graduate School of Medicine, Kyoto, Japan; 5 Technology and Development Team for Mouse Phenotype Analysis, Japan Mouse Clinic, RIKEN BioResource Center, Tsukuba, Japan

**Keywords:** NMDA receptor, Grin1, ENU-mutagenesis, Comprehensive behavioral test battery, Psychiatric disorder, ADHD, Schizophrenia

## Abstract

**Background:**

The *Grin1* (glutamate receptor, ionotropic, NMDA1) gene expresses a subunit of N-methyl-D-aspartate (NMDA) receptors that is considered to play an important role in excitatory neurotransmission, synaptic plasticity, and brain development. *Grin1* is a candidate susceptibility gene for neuropsychiatric disorders, including schizophrenia, bipolar disorder, and attention deficit/hyperactivity disorder (ADHD). In our previous study, we examined an *N*-ethyl-*N*-nitrosourea (ENU)-generated mutant mouse strain (*Grin1*^Rgsc174^/*Grin1*^+^) that has a non-synonymous mutation in *Grin1*. These mutant mice showed hyperactivity, increased novelty-seeking to objects, and abnormal social interactions. Therefore, *Grin1*^Rgsc174^/*Grin1*^+^ mice may serve as a potential animal model of neuropsychiatric disorders. However, other behavioral characteristics related to these disorders, such as working memory function and sensorimotor gating, have not been fully explored in these mutant mice. In this study, to further investigate the behavioral phenotypes of *Grin1*^Rgsc174^/*Grin1*^+^ mice, we subjected them to a comprehensive battery of behavioral tests.

**Results:**

There was no significant difference in nociception between *Grin1*^Rgsc174^/*Grin1*^+^ and wild-type mice. The mutants did not display any abnormalities in the Porsolt forced swim and tail suspension tests. We confirmed the previous observations that the locomotor activity of these mutant mice increased in the open field and home cage activity tests. They displayed abnormal anxiety-like behaviors in the light/dark transition and the elevated plus maze tests. Both contextual and cued fear memory were severely deficient in the fear conditioning test. The mutant mice exhibited slightly impaired working memory in the eight-arm radial maze test. The startle amplitude was markedly decreased in *Grin1*^Rgsc174^/*Grin1*^+^ mice, whereas no significant differences between genotypes were detected in the prepulse inhibition (PPI) test. The mutant mice showed no obvious deficits in social behaviors in three different social interaction tests.

**Conclusions:**

This study demonstrated that the *Grin1*^Rgsc174^/*Grin1*^+^ mutation causes abnormal anxiety-like behaviors, a deficiency in fear memory, and a decreased startle amplitude in mice. Although *Grin1*^Rgsc174^/*Grin1*^+^ mice only partially recapitulate symptoms of patients with ADHD, schizophrenia, and bipolar disorder, they may serve as a unique animal model of a certain subpopulation of patients with these disorders.

## Background

*N*-methyl-*D* aspartate (NMDA) receptors are a class of glutamate receptors composed of heteromeric complexes containing an essential *Grin1* subunit and an additional *Grin2A*-*D* or *Grin3A*-*B* subunit [1-3]. NMDA receptors play important roles in excitatory neurotransmission, synaptic plasticity, and brain development [4-8]. The essential *Grin1* subunit is ubiquitously expressed in the central nervous system (CNS) during the embryonic and adult stages of development [2,9]. Dysfunction of glutamate signaling has been proposed to be involved in the etiology of schizophrenia [10-12]. This hypothesis originates from pharmacological evidence that the abuse of NMDA antagonists, such as ketamine and phencyclidine (PCP), causes symptoms typically observed in schizophrenia, including psychosis, social withdrawal, and working memory deficits [13,14]. Moreover, human genetic studies have suggested that *GRIN1*[15-17], *GRIN2B*[18], and *GRIN2D*[19] are related to genetic susceptibility to schizophrenia. Significant associations have also been reported between *GRIN1* and bipolar disorder [20], as well as between *GRIN2B* and ADHD [21].

*Grin1* hypomorphic mice, which express 5–10% of *Grin1* compared to wild-type, show increased locomotor activity and stereotypy [22,23], impaired social [23,24] and sexual behaviors [22], deficits in nest building [22], and decreased PPI [24], all of which are considered to be behavioral abnormalities relevant to schizophrenia [25]. Other mutant mice with targeted point mutations in *Grin1* show increased locomotor activity [26-28], reduced anxiety-like behaviors [27], abnormal social behaviors [29,30], deficits in spatial working memory [31], and decreased PPI [29], indicating that altered functions in *Grin1* cause behavioral phenotypes related to schizophrenia, bipolar disorder, and ADHD. Recently, *Grin1*^Rgsc174^/*Grin1*^+^ mice, a heterozygous mutant strain with a non-synonymous mutation of the C to T transition in exon 18 (R844C in C0 domain) of *Grin1*, were screened in a large-scale *N*-ethyl-*N*-nitrosourea (ENU) mutagenesis project [32]. The homozygous missense mutation (R844C) caused an increased and prolonged calcium influx in cultured cortical neurons after NMDA stimulation [32]. *Grin1*^Rgsc174^/*Grin1*^+^ mice showed increased locomotor activity, novelty-seeking behavior toward objects, and decreased social interactions [32]. The administration of methylphenidate (MPH), a psychostimulant drug that generally leads to increased locomotor activity in mice, paradoxically attenuates locomotor hyperactivity in *Grin1*^Rgsc174^/*Grin1*^+^ mice [32]. This paradoxical calming effect of MPH in *Grin1*^Rgsc174^/*Grin1*^+^ mice is thought to be analogous to the pharmacological response to MPH in ADHD patients, whose hyperactivity, impulsivity, and attention deficits are attenuated with MPH [33,34]. Therefore, *Grin1*^Rgsc174^/*Grin1*^+^ mice, which have an altered function of their NMDA receptors [32], may also serve as a potential animal model of those disorders. However, other behavioral characteristics, including cognitive functions and sensorimotor gating, have not been fully explored in *Grin1*^Rgsc174^/*Grin1*^+^ mice.

Using a comprehensive behavioral test battery, we found several genetically engineered mouse lines that showed abnormal behaviors related to schizophrenia, such as a severe deficit in working memory and increased locomotor activity [35-39]. Among these mutant mice, alpha-calcium/calmodulin-dependent protein kinase II (αCaMKII) heterozygous knockout (KO) mice [35], forebrain-specific Calcineurin KO [36], SNAP-25 knock-in (KI) [37], and Schnurri-2 (Shn-2) KO mice [38] share an “immature dentate gyrus (iDG)” phenotype, in which the molecular and physiological features of the dentate gyrus (DG) granule cells in the hippocampus are similar to those of immature DG granule cells in normal rodents. Recently, iDG-like phenotypes have been found in the hippocampi of postmortem brains in human schizophrenia/bipolar patients, indicating that iDG represents a candidate endophenotype for the etiology of these diseases [40]. In addition, Grin1 binds to αCaMKII through the C0 domain, a C-terminus domain of Grin1 [41], raising the possibility that the mutation in the C0 domain may alter the interaction between Grin1 and αCaMKII. *Grin1*^Rgsc174^/*Grin1*^+^ mice and αCaMKII HKO mice may share some molecular/cellular abnormalities.

In the present study, we subjected *Grin1*^Rgsc174^/*Grin1*^+^ mice to a comprehensive battery of behavioral tests to further investigate their abnormal behaviors related to neuropsychiatric disorders, such as schizophrenia, bipolar disorder, and ADHD. The battery of behavioral tests included the eight-arm radial maze and the prepulse inhibition (PPI) test, which are used to assess working memory function and sensorimotor gating, respectively. We also conducted neurological screens and wire hang, light/dark transition, open field, elevated plus maze, social interaction, rotarod, hot plate, acoustic startle response, Porsolt forced swim, tail suspension, Contextual and cued fear conditioning, and home cage activity tests. Additionally, using quantitative PCR analysis, we investigated whether *Grin1*^Rgsc174^/*Grin1*^+^ mice have a gene expression pattern that could represent the maturation abnormality in DG granule cells [35-38].

## Results

### General health, neurological reflex, and motor coordination/learning in *Grin1*^*Rgsc174*^*/Grin1*^*+*^ mice

**Figure 1:**
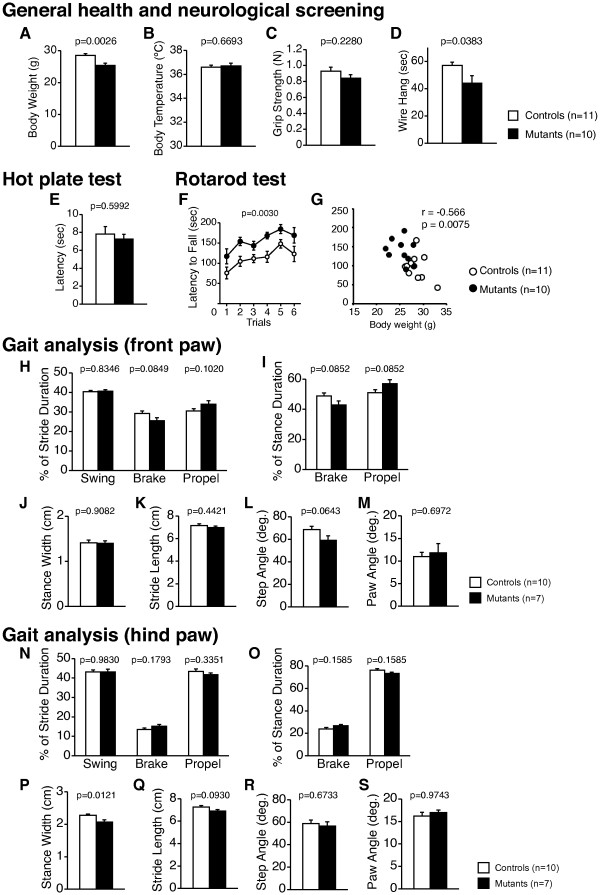
**General health, neurological reflex, nociception, and motor coordination/learning in *Grin1***^**Rgsc174**^**/*Grin1***^**+**^**mice.***Grin1*^Rgsc174^/*Grin1*^+^ mice showed significantly decreased body weight (**A**) and an increased latency to fall in the wire hang test (**D**) compared to wild-type mice. There were no significant differences between the genotypes regarding body temperature (**B**), grip strength (**C**), or latency to the first hind-paw response in the hot plate test (**E**). (**F**) The latency to fall of *Grin1*^Rgsc174^/*Grin1*^+^ mice was significantly increased in the rotarod test. (**G**) Body weight was negatively correlated with rotarod latencies (averaged latency on the 1st day) in *Grin1*^Rgsc174^/*Grin1*^+^ and wild-type mice. In the gait analysis, *Grin1*^Rgsc174^/*Grin1*^+^ mice showed a significant decrease in the stance width of the hind paws (**P**). No significant differences were detected in any other of the following indices of front (**H**-**M**) or hind (**N**-**S**) paw the gait analysis: percentage of stride duration in swing, brake, and propel (**H**, **N**), percentage of stance duration in brake and propel (**I**, **O**), stance width (cm) of front paws (**J**), stride length (cm) (**K**, **Q**), step angle (degree) (**K**, **Q**), and paw angle (degree) (**L**, **R**). The p values indicate the effect of genotype in one-way ANOVA.

No abnormal neurological features, such as whisker twitch or righting reflex, were found in *Grin1*^Rgsc174^/*Grin1*^+^ mice (Table 1). Body weight was significantly reduced (Figure 1A; F_1,19_ = 11.940, p = 0.0026) and latency to fall was significantly decreased in the wire hang test (Figure 1E; F_1,19_=4.956, p=0.0383) in these mutant mice compared to wild-type mice. There were no significant differences in body temperature between the genotypes (Figure 1B; F_1,19_ = 0.188, p = 0.6693), grip strength (Figure 1C; F_1,19_ = 1.552, p = 0.228) or latency to the first hind-paw response in the hot plate test (Figure 1E; F_1,19_ = 0.286, p = 0.5992). *Grin1*^Rgsc174^/*Grin1*^+^ mice showed a significantly longer latency to fall in the accelerating rotarod test compared to wild-type mice (Figure 1F; F_1,19_=11.554, p = 0.030). Previous studies revealed that body weight is negatively correlated with rotarod performance [42,43]. A similar negative correlation was observed in *Grin1*^Rgsc174^/*Grin1*^+^ mice (Figure 1G; body weight vs. average latency (1st day); r = −0.566, p = 0.0065). Analysis of covariance (ANCOVA) with body weight as a covariate found no significant effect of genotype on latency to fall (F_1,19_ = 0.026, p = 0.8730), indicating that the seemingly improved rotarod performance may reflect the reduced body weight of *Grin1*^Rgsc174^/*Grin1*^+^ mice. In the gait analysis, *Grin1*^Rgsc174^/*Grin1*^+^ mice exhibited a significant narrowed stance width of the hind paws (width between hind limbs) compared with wild-type mice (Figure 1O, F_1,15_ = 8.135, p= 0.0121). A previous study reported that the stance width was widened in rats with injured spinal cords and became narrowed during injury recovery after a locomotor training paradigm [44]; therefore, the narrowed stance width in *Grin1*^Rgsc174^/*Grin1*^+^ mice may represent improved motor coordination. No significant differences were detected between genotypes in any other indices of the gait analyses (Figure 1G-R; Figure 1O, F_1,15_ = 8.135, p= 0.0121). These observations indicate that there are no clear deficits in nociception, neuromuscular strength, or motor coordination/learning in *Grin1*^Rgsc174^/*Grin1*^+^ mice.

**Table 1:** **Neurological reflexes in *Grin1***^**Rgsc174**^/***Grin1***^+^**mice**

	**Wild-type**	***Grin1***^**Rgsc174**^**/*Grin1***^**+**^
Coat state (% with normal coat state)	100	100
Ear twitch (% with quick response)	100	100
Whisker (% with)	100	100
Whisker twitch (% with normal response)	100	100
Righting reflex (% with normal response)	100	100
Key jangling (% with normal response)	100	100
Reaching (% with normal response)	100	100

Wild-type mice, n = 10; *Grin1*^Rgsc174^/*Grin1*^+^ mice, n = 11.

### Increased locomotor activity in *Grin1*^Rgsc174^/*Grin1*^+^ mice

**Figure 2:**
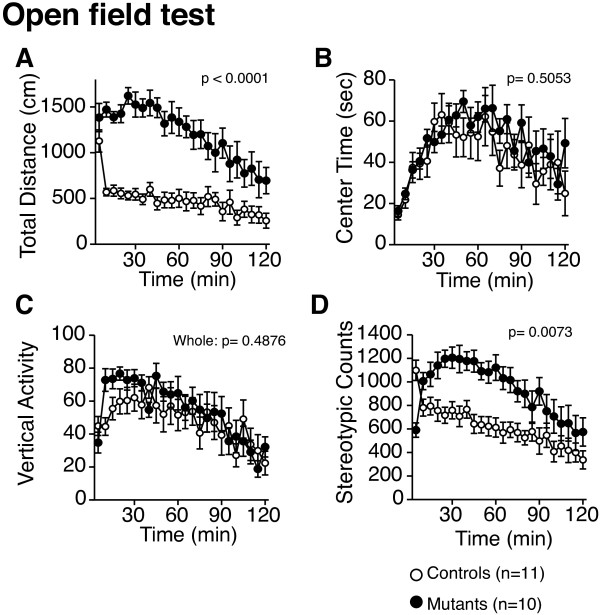
(**A**) **Increased locomotor activity of *Grin1***^**Rgsc174**^/***Grin1***^+^**mice.** In the open field test, the total traveled distance was significantly increased in *Grin1*^Rgsc174^/*Grin1*^+^ mice. (**B**) There was no significant difference between the genotypes in the time spent in the center of the compartment. (**C**) Significant increases were detected in the vertical activity during the earlier portion (0–30 min) of the trials, but not over the total time or in during the later portion (30–120 min) of the trials. (**D**) Stereotypy was significantly increased in *Grin1*^Rgsc174^/*Grin1*^+^ mice. The p values indicate the effect of genotype in two-way repeated measures ANOVA.

**Figure 3:**
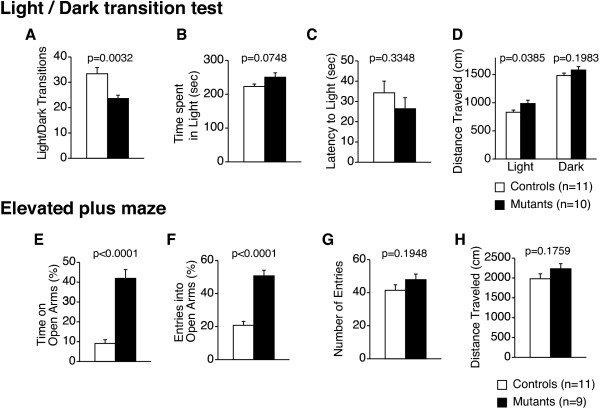
**Abnormal anxiety-like behaviors in *Grin1***^**Rgsc174**^**/*Grin1***^**+**^**mice.** (**A**) In the light/dark transition test, the number of transitions decreased in *Grin1*^Rgsc174^/*Grin1*^+^ mice compared to wild-type mice. No significant differences were detected in the time spent in the light compartment (**B**), latency to enter the light compartment (**C**), or distance traveled in the light/dark compartments (**D**). In the elevated plus maze test, the percentage of time spent in the open arms (**E**) and the percentage of entries into the open arms (**F**) were significantly increased in *Grin1*^Rgsc174^/*Grin1*^+^ mice. There were no significant differences between genotypes in the number of arm entries (**G**) or distance traveled (**H**). The p values indicate the genotype effect in one-way ANOVA.

**Figure 4:**
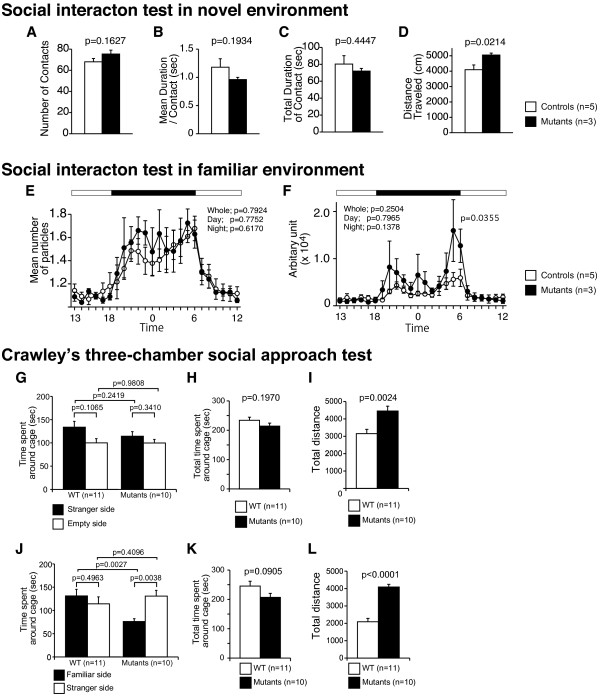
**No obvious deficit in the social interaction of Grin1**^**Rgsc174**^**/Grin1**^**+**^**mice.** In the social interaction test in an open field, there were no significant differences in the number of contacts (**A**), mean duration of each contact (**B**), or total duration of contacts (**C**) between *Grin1*^Rgsc174^/*Grin1*^+^ and wild-type mice. (**D**) The total traveled distance was significantly increased in *Grin1*^Rgsc174^/*Grin1*^+^ mice. In the social interaction test in home cage, there were no significant differences between genotypes in the mean numbers of particles (**E**) or activity levels (**F**). Locomotor activity from 5:00 a.m. to 6:00 a.m. was significantly increased in *Grin1*^Rgsc174^/*Grin1*^+^ mice. (**G**) In the Crawley’s three-chamber social approach test, there was no significant difference in preference between an empty cage and a cage with a stranger mouse, in either wild-type or in *Grin1*^Rgsc174^/*Grin1*^+^ mice. (**H**) No significant differences were detected in total time spent around either the cage with the stranger mouse or the empty cage. (**I**) The distance traveled was significantly increased in *Grin1*^Rgsc174^/*Grin1*^+^ mice. In the social novelty preference test, (**J**) *Grin1*^Rgsc174^/*Grin1*^+^ mice spent a longer time in the cage with a stranger mouse than with the familiar one, but there was no significant difference between genotypes in the time spent in the cage with the stranger mouse. (**K**) There was no significant difference in the total time spent around either cage with the familiar mouse or with the stranger one. (**L**) The total distance traveled by *Grin1*^Rgsc174^/*Grin1*^+^ mice was significantly greater than that of wild-type mice in the social novelty preference test. The p values indicate the effect of genotype in one-way ANOVA, or in two-way repeated measures ANOVA (**E**, **F**) or the paired *t*-tests (**G**, **J**).

In the open field test, the total distance traveled was significantly increased (Figure 2A; F_1,19_ = 31.867, p < 0.0001; genotype × time interaction, *F*_23,437_ = 4.700, p < 0.0001), while no significant increase in time spent in the center area was detected in *Grin1*^Rgsc174^/*Grin1*^+^ mice compared to wild-type mice (Figure 2B; F_1,19_ = 0.461, p = 0.5053). These observations are consistent with a previous study [31]. There was no significant effect of the genotype in vertical activity for 120 min (Figure 2C; F_1,19_ = 0.501, p = 0.4876), although there was a significant genotype × time interaction between genotypes (Figure 2C; *F*_23,437_ = 1.882, p =0.0085). Stereotypic behavior was significantly increased in *Grin1*^Rgsc174^/*Grin1*^+^ mice compared with wild-type mice (Figure 2D; F_1,19_ = 9.015, p = 0.0073). Increased locomotor activity of *Grin1*^Rgsc174^/*Grin1*^+^ mice was detected in other behavioral measures: total distance traveled in a light chamber in the light/dark test (Figure 3D; F_1,19_=4.942, p=0.0385), the social interaction test in a novel environment (Figure 4D; F_1,38_=8.141, p=0.0214), and the Crawley’s sociability (Figure 4I; F_1,19_ = 12.224, p =0.0024) and social novelty preference tests (Figure 4L; F_1,19_ = 61.313, p < 0.0001).

### Abnormal anxiety-like behaviors in *Grin1*^Rgsc174^/*Grin1*^+^ mice

In the light/dark transition test, the number of transitions between chambers was significantly decreased in *Grin1*^Rgsc174^/*Grin1*^+^ mice (Figure 3A; F_1,19_=11.418, p=0.0032). There were no significant differences between genotypes in the time they remained in a light chamber (Figure 3B; F_1,19_=3.554, p=0.0748) or in the first latency to enter the light chamber (Figure 3C; F_1,19_=0.979, p=0.3348). *Grin1*^Rgsc174^/*Grin1*^+^ mice traveled significantly longer distances in the light chamber (Figure 2D; F_1,19_=4.942, p=0.0385), but not in the dark one (Figure 2D; F_1,19_=1.776, p=0.1983). In the elevated plus maze test, the percentage of time spent in the open arms and the percentage of entries into the open arms by *Grin1*^Rgsc174^/*Grin1*^+^ mice were significantly higher than those by wild-type mice (Figure 2E; time spent in the open arms, F_1,18_=54.874, p<0.0001; Figure 2F; entries into the open arms, F_1,18_=55.392, p < 0.0001). There were no significant differences between the genotypes in the total number of entries into the arms (Figure 2G; F_1,18_=1.814, p = 0.1948) or in the distance traveled (Figure 2H; F_1,18_ = 1.985, p = 0.1759). The number of transitions between the chambers decreased in the light/dark transition test, suggesting increased anxiety-like behavior in *Grin1*^Rgsc174^/*Grin1*^+^ mice. On the other hand, the mutant mice displayed a higher percentage of time spent in open arms and a higher percentage of entries into the open arms in the elevated plus maze test, which are generally interpreted as decreased anxiety-like behaviors. Thus, apparently opposite anxiety-like behaviors were observed between the light/dark transition test and elevated plus maze test. For a more detailed interpretation of these results, see the Discussion section.

### No abnormalities in the Porsolt forced swim or tail suspension tests in *Grin1*^Rgsc174^/*Grin1*^+^ mice

**Figure 5:**
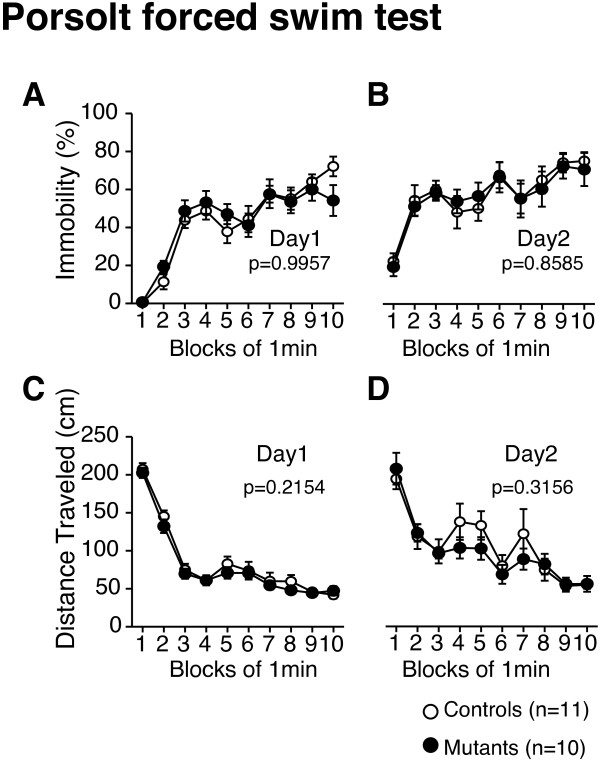
**Normal depression-like behavior in *Grin1***^**Rgsc174**^**/*Grin1***^**+ **^**mice.** In the Porsolt forced swim test, there were no significant differences in the percentage of immobility time at day 1 (**A**) or day 2 (**B**), or in the distance traveled at day 1 (**C**) or day 2 (**D**) between *Grin1*^Rgsc174^/*Grin1*^+^ mice and wild-type mice. The p values indicate the effect of genotype in two-way repeated measures ANOVA. Bars indicate the means ± standard errors of the mean.

There were no significant differences between the *Grin1*^Rgsc174^/*Grin1*^+^ and wild-type mice in immobility on day 1 (Figure 5A; genotype effect, F_1,19_ = 2.949 × 10^-5^, p = 0.9957; genotype × time interaction, *F*_9,171_ = 1.099, p = 0.3659) or day 2 (Figure 5B; genotype effect, F_1,19_ = 0.033, p = 0.8585; genotype × time interaction, *F*_9,171_ = 0.180, p = 0.9959), or in distance traveled on day 1 (Figure 5C; genotype effect, F_1,19_ = 1.642, p = 0.2154) or day 2 (Figure 5D; genotype effect, F_1,19_ = 1.062, p = 0.3156) in the Porsolt forced swim test. In the tail suspension test, there was no significant difference in immobility between the genotypes (Table 2).

**Table 2:** **Tail suspension and prepulse inhibition tests of *Grin1***^**Rgsc174**^/***Grin1***^+^**mice**

**Behavioral test**		**Wild-type**	**Mutant**	**F value(degree of freedom)**	**p value**
Tail suspension test					
	Immobility	15.3 (±3.8)	30.3 (±8.3)	F_1,14_ = 3.152	0.098
Prepulse inhibition test					
	Startle stimulus/Prepulse (dB)				
	110/74	−0.8 (±11.6)	−4.1 (±12.3)	F_1,18_ = 0.357	0.558
	110/78	−12.8 (±14.9)	−4.9 (±17.6)		
	120/74	−14.3 (±6.5)	−14.3 (±10.5)	F_1,18_ = 1.589	0.224
	120/78	−6.1 (±9.5)	−6.1 (±9.6)		

### No obvious deficit in the social interactions of *Grin1*^Rgsc174^/*Grin1*^+^ mice

In the social interaction test conducted in a novel environment, there were no significant differences between the *Grin1*^Rgsc174^/*Grin1*^+^ and wild-type mice in the number of contacts (Figure 4A; F_1,8_ = 2.364, p = 0.1627), mean duration per contact (Figure 4B; F_1,8_ = 2.017, p = 0.1934), or total duration of contact (Figure 4C; F_1,8_ = 0.646, p = 0.4447). These results are inconsistent with the previous results indicating that the total interaction time in *Grin1*^Rgsc174^/*Grin1*^+^ mice had significantly decreased compared to that in wild-type mice in an open field during a social interaction test [31]. In the test of social behaviors in home cage, no significant difference was detected in social interaction between *Grin1*^Rgsc174^/*Grin1*^+^ and wild-type mice (Figure 4E; whole period, F_1,6_ = 0.076, p = 0.7924; light period, F_1,6_ = 0.089, p=0.7752; dark period, F_1,6_ = 0.278, p=0.6170). There was no significant effect of genotype on locomotor activity, which was quantified as the number of pixels changed between each pair of successive frames in home cage (Figure 4F; whole period, F_1,6_ = 2.101, p = 0.1974; day period, F_1,6_ = 0.005, p = 0.9480; night period, F_1,6_ = 3.843, p=0.0976), although there was a significant genotype × time interaction between the genotypes (Figure 2C; *F*_23,437_ = 1.882, p =0.0085). Given that the mutant mice showed significantly increased locomotor activity in their home cages in the previous observations [31] and in one-sided testing of the present study (p= 0.0488), the non-significant genotype effect was most likely caused by the small number of pairs (Wild-type, N=5; Mutant mice, N=3). In fact, *Grin1*^Rgsc174^/*Grin1*^+^ mice displayed apparently increased locomotor activity throughout the night period, and this activity was significantly increased during the time period from 5:00 a.m. to 6:00 a.m. (Figure 4F; F_1,6_ = 7.297, p=0.0355) in their home cage. In the Crawley’s three-chamber social approach test, there was no significant difference in preference between the empty cage and the cage with a stranger mouse, either in wild-type or *Grin1*^Rgsc174^/*Grin1*^+^ mice (Figure 4G; time spent around the cages; stranger cage vs. empty cage; paired *t*-test; wild-type mice, t_10_= 1.774, p= 0.1065; *Grin1*^Rgsc174^/*Grin1*^+^ mice, t_9_ = 1.005, p = 0.3410). No significant difference between genotypes was detected in the total time spent around the empty chamber or the chambers of the stranger mouse (Figure 4H; genotype, F_1,19_ = 1.788, p = 0.1970). In the social novelty preference test, *Grin1*^Rgsc174^/*Grin1*^+^ mice spent more time in the cage with a novel (stranger) mouse compared to the time spent in the cage with the familiar mouse (the first, already-investigated mouse), whereas wild-type mice did not show a preference between the familiar mouse and a stranger mouse (Figure 4J; time spent in cages (familiar vs. stranger); paired *t*-test; *Grin1*^Rgsc174^/*Grin1*^+^ mice, t_9_ = 3.877, p = 0.0038; wild-type mice, t_10_ = −0.706, p = 0.4963). Similar results were obtained in the time spent around the chambers (familiar side vs. stranger side, paired *t*-test; wild-type mice, t_10_ =−0.451, p=0.6616; *Grin1*^Rgsc174^/*Grin1*^+^ mice, t_9_ =3.721, p=0.0048). In comparison with wild-type mice, *Grin1*^Rgsc174^/*Grin1*^+^ mice spent significantly less time in the cage with the familiar mouse (Figure 4J; genotype, F_1,19_ = 11.847, p = 0.0027), while no significant difference between the genotypes was detected in time spent in the cage with a novel mouse (Figure 4J; genotype, F_1,19_ = 0.711, p = 0.4096). These observations suggest that social novelty preference is increased in *Grin1*^Rgsc174^/*Grin1*^+^ mice. There was no significant difference between the genotypes in the total time spent around both the cages of the familiar and of the stranger mice (Figure 4K; genotype, F_1,19_ = 3.180, p = 0.0905). There were no obvious impairments in the *Grin1*^Rgsc174^/*Grin1*^+^ mice in three different tests for social behavior.

### Severely impaired fear memory in *Grin1*^Rgsc174^/*Grin1*^+^ mice

**Figure 6:**
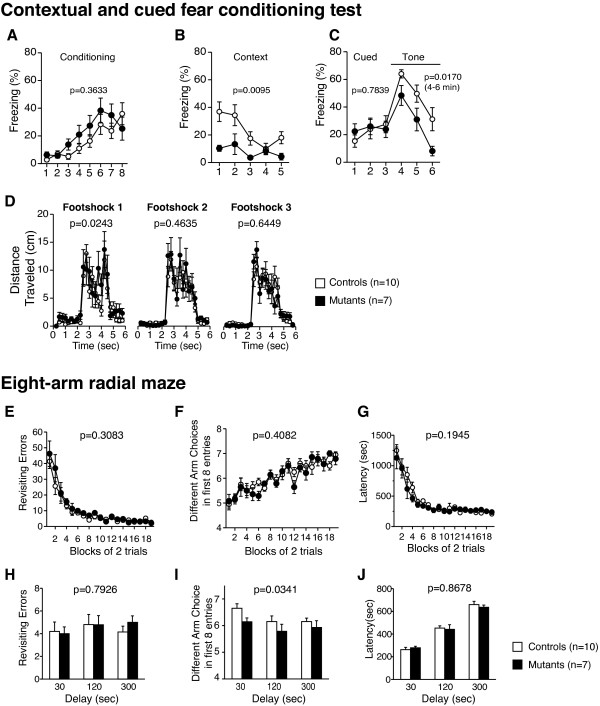
**Severely impaired fear memory and mild deficits in the spatial working memory in *Grin1***^**Rgsc174**^/***Grin1***^+^**mice.** (**A**) There were no significant differences between the genotypes in the percentage of time freezing during conditioning in the contextual and cued fear conditioning tests. *Grin1*^Rgsc174^/*Grin1*^+^ mice displayed a decreased percentage of time freezing in the contextual (**B**) and cued tests (**C**) compared to wild-type mice. *Grin1*^Rgsc174^/*Grin1*^+^ mice showed an increase in distance traveled immediately after footshock 1, but not after footshocks 2 or 3 in the training phase (**D**). In the eight-arm radial maze, there was no significant difference between *Grin1*^Rgsc174^/*Grin1*^+^ and wild-type mice in the number of revisiting errors (**E**), different arm choices (**F**), or latency (**G**) in the first eight entries during the test session without delay (1–19 blocks). No significant difference between the genotypes was detected in the number of revisiting errors (**H**), or latency (**J**) in the later session (20–22 blocks) with delays (30, 120, and 300 sec). The mutant mice showed a significantly lower number of different arm choices during the first eight entries in the trials with delay (**I**). The p values indicate the genotype effect in two-way repeated measures ANOVA.

In the contextual and cued fear conditioning test, there was no significant difference in freezing between genotypes in the conditioning phase (Figure 6A; genotype effect, F_1,15_ = 0.879, p = 0.3633; genotype × time interaction, *F*_7,105_ = 1.701, p = 0.1165). Contextual freezing at 1 day after training was significantly decreased in *Grin1*^Rgsc174^/*Grin1*^+^ mice compared to wild-type mice (Figure 6B; genotype effect, F_1,15_=8.848, p = 0.0095). In the altered context, although there was no significant difference in freezing during the pre-tone period between genotypes (Figure 6C; genotype effect, F_1,15_ = 0.078, p = 0.7839), freezing during the tone period was significantly lower than in wild-type mice (Figure 6C; genotype effect, F_1,15_ = 7.206, p = 0.017). *Grin1*^Rgsc174^/*Grin1*^+^ mice showed a significant increase in distance traveled immediately after the first footshock compared to wild-type mice, and no significant differences were detected in these measurements after the second or third footshocks (Figure 6D; Footshock 1, F_1,15_ = 6.270, p = 0.0243; Footshock 2, F_1,15_ = 0.566, p = 0.4635; Footshock 3, F_1,15_ = 0.221, p = 0.6449). These findings demonstrate that both contextual and cued fear memory are impaired in *Grin1*^Rgsc174^/*Grin1*^+^ mice.

### Moderately impaired spatial working memory in *Grin1*^Rgsc174^/*Grin1*^+^ mice

In the eight-arm radial maze test, there were no significant differences in the number of revisiting errors during the trials without delays (1–19 blocks, Figure 6E; genotype effect, F_1, 15_ =1.112, p = 0.3083; genotype × trials interaction, F_18,270_ = 0.647, p = 0.8602) or during the trials with delay (20–22 blocks Figure 6H; genotype effect, F_1,15_ =0.072, p = 0.7926; genotype × trials interaction, F_2,30_ = 0.398, p = 0.6753) between the *Grin1*^Rgsc174^/*Grin1*^+^ and wild-type mice. No significant difference was detected in the number of different arm choices during the first eight entries in the trials without delays (1–19 blocks, Figure 6F; genotype effect, F_1,15_= 0.724, p = 0.4082; genotype × trials interaction, F_18,270_ = 0.837, p = 0.6562). To increase the difficulty of the task, a delay period (30 sec, 2 min, and 5 min) was provided after four pellets had been taken by confining the mice to the center platform in the 20th, 21st, and 22nd blocks of the trials. *Grin1*^Rgsc174^/*Grin1*^+^ mice showed a significantly lower number of different arm choices during the first 8 entries in the trials with delay (Figure 6I; genotype effect, F_1,15_ =5.431, p=0.0341; genotype × trials with delay interaction, F_2,30_ = 0.251, p=0.7792). No significant difference was detected in the latency to obtain all pellets between *Grin1*^Rgsc174^/*Grin1*^+^ and wild-type mice (Figure 6G; without delay, genotype effect, F_1,15_ = 1.833, p = 0.1945; genotype × trials interaction, F_18,270_ = 1.143, p = 0.2611; Figure 6J; with delay, genotype effect, F_1,15_= 0.370, p = 0.8678; genotype × trials interaction, F_2,30_ = 0.370, p = 0.8678). The results of the eight-arm radial maze test suggest moderately impaired spatial working memory in *Grin1*^Rgsc174^/*Grin1*^+^ mice. However, it is possible that the increased locomotor activity causes the mild performance deficit to become a confounding factor.

### *Decreased startle response of Grin1*^Rgsc174^/*Grin1*^+^*mice*

**Figure 7:**
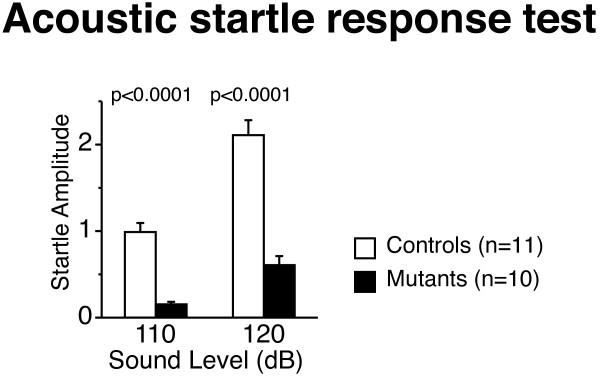
**Decreased startle response in *Grin1***^**Rgsc174**^**/*Grin1***^**+**^**mice.***Grin1*^Rgsc174^/*Grin1*^+^ mice had significantly lower startle amplitude than wild-type mice at both 110 dB and 120 dB. The p values indicate the effect of genotype in one-way ANOVA.

*Grin1*^Rgsc174^/*Grin1*^+^ mice had markedly lower startle amplitude than wild-type mice at both 110 dB and 120 dB (Figure 7A; genotype effect, 110 dB: F_1,18_ = 57.464, p < 0.0001, 120: F_1,18_=83.542, p < 0.0001). We did not detect significant differences in PPI between the genotypes (Table 2).

### Expression pattern of maturation markers of DG neurons in *Grin1*^Rgsc174^/*Grin1*^+^ mice

**Figure 8:**
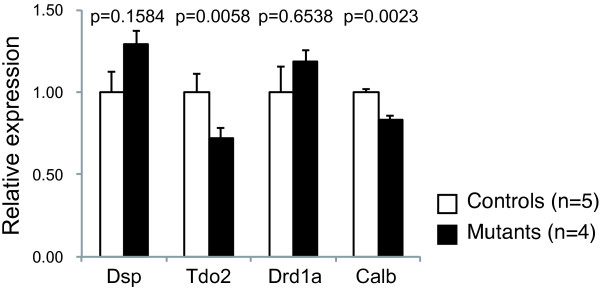
**Expression pattern of maturation markers of DG neurons in *Grin1***^**Rgsc174**^**/*Grin1***^**+**^**mice.** The expression of *Tdo2* and *Calb* was significantly reduced in *Grin1*^Rgsc174^/*Grin1*^+^ mice compared to those in wild-type mice. There were no significant differences between the genotypes in the expression of *Dsp* or *Drd1a*. The p values indicate the effect of genotype in one-way ANOVA.

To address whether *Grin1*^Rgsc174^/*Grin1*^+^ mice show maturation abnormalities in dentate granule cells, the expression patterns of markers for “immature dentate gyrus (iDG)”, upregulation of *dopamine receptor D1A* (*Drd1a*) and downregulation of *desmoplakin* (*Dsp*), *tryptophan 2*,*3*-*dioxygenase* (*Tdo2*), and *Calbindin*-*28k* (*Calb1*), were assessed by quantitative RT-PCR using total RNA extracted from the hippocampus. The expression of *Tdo2* and *Calb1* was slightly but significantly reduced in the mutant mice compared to wild-type mice (Figure 8; *Tdo2*, p = 0.006; *Calb1*, p = 0.002). There were no significant differences between *Grin1*^Rgsc174^/*Grin1*^+^ and wild-type mice in the expressions of *Dsp* or *Drd1a* (Figure 8; *Dsp*, p = 0.197; *Drd1a*, p=0.652). In *Grin1*^Rgsc174^/*Grin1*^+^ mice, two out of the four marker genes showed iDG-specific gene expression patterns, suggesting that they may partially display the iDG phenotype.

## Discussion

In the present study, we used a comprehensive battery of behavioral tests to analyze the physical and behavioral profiles of an ENU-generated mutant mouse strain (*Grin1*^Rgsc174^/*Grin1*^+^) with a non-synonymous mutation in *Grin1*. *Grin1*^Rgsc174^/*Grin1*^+^ mice exhibited increased locomotor activity, abnormal anxiety-like behaviors, severe deficits in fear memory, moderately impaired spatial working memory, and decreased acoustic startle responses. No obvious deficits were observed in neuromuscular strength or social behaviors in *Grin1*^Rgsc174^/*Grin1*^+^ mice. There were no significant differences between genotypes in the Porsolt forced swim, tail suspension, or PPI tests.

NMDA receptors are thought to be involved in fear memory [45-47]. Pharmacological studies have shown that the administration of NMDA antagonists blocks synaptic transmission in the amygdala and the acquisition of fear memory [47-50]. Studies using inducible and reversible *Grin1* KO mice also demonstrated that the expression of *Grin1* in the forebrain is necessary for the consolidation [51] and preservation of remote memory [52] in the fear conditioning test. An increased and prolonged Ca^2+^ influx after activation of the NMDA receptor was observed in cultured cells derived from the cortices of *Grin1*^Rgsc174^/ *Grin1*^Rgsc174^, but not in *Grin1*^Rgsc174^/*Grin1*^+^ mice [32]. However, *in vivo*, it is possible that NMDA receptor signaling is also dysregulated also in *Grin1*^Rgsc174^/*Grin1*^+^ mice. Severely impaired contextual and cued fear memory may be caused by the perturbation of signaling via NMDA receptors in the amygdala and/or forebrain in *Grin1*^Rgsc174^/*Grin1*^+^ mice. Further studies are required to elucidate the precise mechanisms by which fear memory is impaired in *Grin1*^Rgsc174^/*Grin1*^+^ mice.

*Grin1*^Rgsc174^/*Grin1*^+^ mice exhibited increased time spent in the open arms and a greater number of entries into the open arms in the elevated plus maze test, which is generally interpreted as a decrease in anxiety-like behavior. In contrast, in the light/dark transition test, *Grin1*^Rgsc174^/*Grin1*^+^ mice displayed a decreased number of transitions between the light and dark chambers, which is a well-validated index of anxiety-like behavior [53]. Forebrain-specific Calcineurin KO mice [54], pituitary adenylate cyclase-activating polypeptide (PACAP) KO mice [39], and Shn-2 KO mice [38] also showed these apparently conflicting abnormal behaviors: smaller number of transitions and/or prolonged latency in the light box in the light/dark transition test, and increases in time spent in the open arms and/or in the number of entries into the open arms in the elevated plus maze test. This pattern of abnormalities has been interpreted in previous studies to reflect an elevated panic-like escape response to stress and/or a higher level of anxiety [54-56]. The plasma corticosterone (CORT) level is a recognized measure of sensitivity to stress [57]. Shn-2 KO mice showed increased plasma CORT levels after the elevated plus maze test, which were significantly higher than those in the wild-type mice [58]. In *Grin1*^Rgsc174^/*Grin1*^+^ mice, increases in locomotor activity were not observed in the elevated plus maze test or in the first period (0–5 min) of the open field test, although increased locomotor activity, a robust behavioral phenotype, was detected in the later period of the open field, home cage, and light/dark transition tests in *Grin1*^Rgsc174^/*Grin1*^+^ mice. These observations suggest that anxiety is possibly elevated in *Grin1*^Rgsc174^/*Grin1*^+^ mice, which might cause suppression of locomotor hyperactivity under a stressful and/or novel situation. Further studies are needed to determine whether anxiety is increased or decreased in *Grin1*^Rgsc174^/*Grin1*^+^ mice. For example, in the elevated plus maze test, it would be interesting to evaluate whether administration of anxiolytic drugs suppress the increased time spent in open arms, reflecting an elevated panic-like escape response or a decreased anxiety-like behavior in the mutant mice. In addition, anxiety could be evaluated through quantification of serum corticosterone levels, which increase in response to stress, or of c-fos expression in the brain regions that are thought to play a role in emotional processing, including the amygdala, midline thalamic nuclei, several medial hypothalamic nuclei, and dorsal raphe nucleus [58-60]. Anxiety-like behaviors could be affected by prior tests [61,62], and the order of tests employed in the present study, might have caused the apparently discrepant results between the light/dark transition and elevated plus maze tests. It is possible that it takes a long time for the mutant mice to become familiar with the behavioral test battery. Therefore, the mutant mice apparently showed increased anxiety-like behavior in light/dark transition test performed as the first test in our behavioral test battery, and the mutant mice might display intrinsically decreased anxiety-like behavior in the elevated plus test that was conducted in the later part of the test battery. To test this possibility, it would be important that the light/dark transition and elevated plus maze tests are performed in a varied order, or the elevated plus maze test is conducted first in the test battery.

There were no significant differences in social interaction tests in either the open field or the home cage in the present study, which might be due to the small number of pairs used in these tests (wild-type, N=5; mutant, N=3). The social novelty preference in Crawley’s three-chamber test increased in *Grin1*^Rgsc174^/*Grin1*^+^ mice. The wild-type mice exhibited no significant preference for social novelty. This result is sometimes observed with the protocol used for the present study, in which the mice are habituated to the apparatus the day before the social novelty preference test. When habituation is accomplished just before the test, social novelty preferences are detected in wild-type mice (data not shown). It would be of interest to perform the social novelty preference test with the modified protocol. Because increased novelty-seeking behavior toward objects was also observed in these mutant mice [32], it is possible that the increased social novelty preference observed here may reflect an enhanced general novelty-seeking tendency of the mice. *Grin1*^Rgsc174^/*Grin1*^+^ mice did not show obvious abnormal social behaviors that have been observed in other mouse lines that exhibit behavioral abnormalities related to schizophrenia [25]. These observations do not suggest that *Grin1*^Rgsc174^/*Grin1*^+^ mice recapitulate social withdrawal, which is a negative symptom of schizophrenia.

Mice lacking *Grin2A*, a subtype of one of the NMDA subunits, display mildly weakened neuromuscular strength in the wire-hang and balance beam tests and normal performance in the grip strength test [63]. The decreased latency to fall observed in the accelerating rotarod test in *Grin2A* KO mice suggests that motor coordination/learning is impaired in these mutant mice [64]. In our study, a trend similar to that observed for *Grin2A* KO mice was detected in the test of neuromuscular strength in *Grin1*^Rgsc174^/*Grin1*^+^ mice: a short latency to fall in the wire hang test and a normal performance in the grip strength test. In contrast, the latency to fall of *Grin1*^Rgsc174^/*Grin1*^+^ mice increased in the rotarod test. The lower body weight of *Grin1*^Rgsc174^/*Grin1*^+^ mice might explain the increased performance in this test. Consistent with this hypothesis, an analysis of covariance (ANCOVA) using body weight as a covariate found no significant effect of genotype on rotarod latencies, indicating that motor coordination/learning is normal in *Grin1*^Rgsc174^/*Grin1*^+^ mice. It is possible that the lower performance in the wire hang test was caused by increased locomotor activity in *Grin1*^Rgsc174^/*Grin1*^+^ mice. Additionally, the narrowed stance width of the hind paws in *Grin1*^Rgsc174^/*Grin1*^+^ mice possibly reflects improved postural adjustments for stability, as indicated in the case of injury recovery after a locomotor training paradigm [44]. These observations indicate that *Grin1*^Rgsc174^/*Grin1*^+^ mice may not have deficits in neuromuscular strength or motor coordination/learning.

The number of transitions of *Grin1*^Rgsc174^/*Grin1*^+^ mice decreased in the light/dark transition test in this study but not in the previous study [32]. Decreased social behavior was observed in an open field of the previous study, but the present study failed to detect any impairment of social behaviors in a similar situation, in home cage, or in the Crawley’s three-chamber social approach test. However, the social novelty preference of *Grin1*^Rgsc174^/*Grin1*^+^ mice increased in the Crawley’s three-chamber test. These discrepancies might be due to differences in the behavioral test methods and the experimental conditions between the previous and present studies, such as the apparatuses, protocols, experimenter, age of subjects, number of cage mates, and experiences of subjects. The previous study used experimentally naïve mice in each behavioral test [32].

*Grin1* mutant mouse strains with knockdown alleles or point mutations show unique sets of abnormal behaviors (Table 3). *Grin1*^Rgsc174^/*Grin1*^+^ mice displayed a few unique behavioral abnormalities that have not been observed in other strains of *Grin1* mutant mice. The acoustic startle response markedly decreased in our mice, while it increased in *Grin1* hypomorphic mice [24] and in *Grin1*(D481N)/ *Grin1*^+^ mice [31]. Increased locomotor activity was shared with the majority of other *Grin1* mutant strains, *Grin1*(N598Q)/*Grin1* mice [64], *Grin1*(N598R)/*Grin1*[65] mice, *Grin1* hypomorphic mice [22], and *Grin1*^Rgsc174^/*Grin1*^+^ mice. *Grin* hypomorphic [22], *Grin1*(D481N)/*Grin1*^+^[30], and our mice consistently displayed increased time spent in open arms in the elevated plus maze or zero maze tests, suggesting decreased anxiety-like behavior in these mice. However, in the light/dark transition test, there was no difference in the number of transitions in *Grin1*(D481N)/*Grin1*^+^[31], and that of *Grin1*^Rgsc174^/*Grin1*^+^ mice was decreased. In the open-field test, while *Grin1*(D481N)/*Grin1*^+^ mice [31] and *Grin* hypomorphic [23] showed increased time spent in the center area, there was no increase in *Grin1*^Rgsc174^/*Grin1*^+^ mice. There were no significant difference between *Grin1*^Rgsc174^/*Grin1*^+^ and wild-type mice in the PPI test, whereas PPI was decreased in *Grin1* hypomorphic mice [24] and *Grin1*(S897A)/*Grin1*(S897A) mice [29]. Regarding social behavior, our mice did not show the obvious decreased social behavior observed in *Grin1*(S897A)/*Grin1*(S897A) [29] and *Grin* hypomorphic mice [22,24]. The unique profile of behavioral abnormalities in each *Grin1* mutant strain could be due to differences in the molecular and/or cellular functions of *Grin1*, e.g., decreased/increased calcium influx, disrupted glycine binding, etc. (Table 3). Alternatively, the behavioral profile may reflect differences in experimental conditions, such as genetic background, age of subjects, apparatuses, and protocols.

**Table 3:** **Mutations in*Grin1*cause distinctive behavioral abnormalities**

		***Grin1***^**Rgsc174**^		***Grin1***^**tm1**.**1Ese**^	***Grin1***^**tm1**.**1Phs**^		***Grin1***^**tm1**.**1Slab**^	***Grin1***^**tm1Blt**^	***Grin1***^**tm2Blt**^	***Grin1***^**tm1Bhk**^
	**Mutation**	R844C		S897A	N598Q		N598R	D481N	K483Q	Insertion of a neomycin cassette (hypomorphic)
	**Site**/**Domain**	C0 domain (interacting with CaMKII, calmodulin, and alpha-actinin)		Phosphorylation site for PKA	Critical channel site		Critical channel site	Glycine binding site	Glycine binding site	Intron 20
	**Genotype**	*Grin1*^Rgsc174^/*Grin1*^+^	*Grin1*^Rgsc174^/*Grin1*^*Rgsc174*^	*Grin1*^tm1.1Ese^/*Grin1*^tm1.1Ese^	*Grin1*^tm1.1Phs^/*Grin1*^+^	*Grin1*^tm1.1Phs^/*Grin1*^*tm1*.*1Phs*^	*Grin1*^tm1.1Slab^/*Grin1*^+^	*Grin1*^tm1Blt^/*Grin1*^+^	*Grin1*^tm2Blt^/*Grin1*^*tm2Blt*^	*Grin1*^tm1Bhk^/*Grin1*^*tm1Bhk*^
	**Effects on receptor function**	ND	Increased Ca^2+^ influx [32]	No phosphorylation at S897[29]	No obvious defects [64]	Reduced Ca^2+^ permeability; Altered voltage-dependent Mg^2+^ block [64]	Reduced Ca^2+^ permeability; Lower mean current amplitude mediated by NMDA receptor; Strong reduction of the Mg^2+^ block [65]	5-fold reduction in receptor glycine affinity; Normal glutamate affinity [31]	86-fold reduction in receptor glycine affinity; Normal glutamate affinity [31]	ND
	**Cellular**/**tissue**-**level effects**	Enhanced c-Fos immunoreactivity in the prelimbic cortex [32]	ND	Abnormal glutamate mediated receptor currents; Reduced AMPA-mediated synaptic currents, reduced long-term potentiation [29]	Normal CA3/CA1 synapse LTP [64]	ND	Normal whisker barrel formation in the primary somatosensory cortex [65]	Decreased susceptibility to pharmacologically induced seizures; Reduced LTP [31]	Reduction in glycine concentration-dependent Ca^2+^ influx	ND
	**Effects on expression level**	Normal [32]	ND	Decreased at synapse [29]	Normal	Normal	Normal	Large increase in Cerebellum; Grin2B shows large increase in Cortex, Striatum, and Cerebrum [31]	ND	Reduced expression to ~10% of normal levels [22]
	**Physical phenotype**	Decreased body weight [present study, 32]	Premature death (Embryonic 17th day to 4 weeks) [32]	ND	Premature death [64]	Death ~1 hr after birth; respiratory distress; no feeding [64]	Perinatal death ~6 hr from birth [65]	Normal development [31]	Postnatal lethality, decreased body weight	Decreased body weight [22]; Reduced male fertility [22]
	**Locomotor activity**	Increased in OF and HC [present study, 32]	ND	Normal in HC [29]	Increased before delivery (Pregnant females) [64]	ND	Increased (Newborn) [65]	ND	ND	Increased during habituation in OF [22,23]; Normal in HC [23]
	**Social behavior**	Shorter interaction with other subject in OF [32]; Increased social novelty preference in CSI [present study]	ND	Abnormal social investigation [29]	ND	ND	ND	Social approach deficit [30]	ND	Social withdrawal, escape behaviors, reduced social investigation in RI [22]; Lower approach toward the stimulus mouse in TCS [23]; No preference for unfamiliar mouse in SA [24];
**Behavioral phenotypes**	**Anxiety**-**like behavior**	Decreased transition in LD; Increased time spent in open arm in EP [present study]	ND	ND	ND	ND	ND	Increased spent time in open arm of EP [30]; Increased time spent in center of OF [31]; Increased time exploring object [30]; No difference in LD [31];	ND	Increased time spent in open arm in EZ [23]; Increased time spent in the central zone in OF [23]
	**Startle response**	Decreased [present study]	ND	Normal [29]	ND	ND	ND	Increased [31]	ND	Increased [24]
	**Prepulse inhibition**	No significant difference between genotypes	ND	Decreased [29]	ND	ND	ND	Normal	ND	Decreased [24]; Increased amplitudes for auditory and visual ERPs [23]
	**Working memory**	No obvious deficit	ND	ND	ND	ND	ND	Abnormal spatial learning in MWM [31]	ND	ND
	**Fear memory**	Decreased	ND	ND	ND	ND	ND	ND	ND	ND
	**Other behavioral phenotypes**	Increased exploration [32]; Normal depression-like behavior in TS and PS [present study]	ND	ND	Abnormal nest building; Abnormal maternal nurturing (abnormal maternal grooming, abnormal pup retrieval, pup cannibalization) [64]	ND	ND	Impaired motor coordination in horizontal wire test [31]	No suckling reflex	Potential impairments in olfaction [76]; Reduced nest building behavior [23]
	**Background**	C57BL/6J	C57BL/6J	C57BL/6	129S1/Sv × 129X1/SvJ × C57BL/6		129P2/OlaHsd × C57BL/6J	129P2/OlaHsd × C57BL/6	129P2/OlaHsd × C57BL/6	129P2/OlaHsd × C57BL/6 × DBA/2

Human genetic studies have suggested that some subunits of NMDA receptors are associated with psychiatric disorders, such as schizophrenia [15,17-19,66], bipolar disorder [20], and ADHD [21]. *Grin1*^Rgsc174^/*Grin1*^+^ mice showed abnormal behaviors related to these disorders, including increased locomotor activity, severely impaired fear memory, mild deficit in working memory, and abnormal anxiety-like behavior. Psychomotor agitation is a symptom of schizophrenia, and psychostimulants that induce schizophrenic behaviors in healthy individuals increase locomotor activity in rodents [67]. An increase of locomotor activity in *Grin1*^Rgsc174^/*Grin1*^+^ mice also seems to be a trait related to ADHD. Additionally, MPH administration paradoxically attenuates locomotor hyperactivity in *Grin1*^Rgsc174^/*Grin1*^+^ mice [31]. Patients with schizophrenia display various forms of memory defects, including impaired working and episodic memory [68,69]. It is possible that deficits in the fear memory of *Grin1*^Rgsc174^/*Grin1*^+^ mice represent an aspect of the cognitive impairment of schizophrenia. Although increased anxiety is not a core symptom of schizophrenia, an epidemiological association has been suggested between anxiety and schizophrenia [70,71]. Abnormal anxiety-like behaviors are found in other animal models of schizophrenia, such as forebrain-specific calcineurin KO [54], *Mus musculus* microtubule-associated protein 6 (Mtap6 or STOP) KO [72], Shn-2 KO [38], or SNAP-25 KI [37] mice. There were no obvious impairments in social behavior in *Grin1*^Rgsc174^/*Grin1*^+^ mice or significant differences in PPI between the genotypes. Reduced acoustic startle response was observed in *Grin1*^Rgsc174^/*Grin1*^+^ mice. This may be caused by deficits in emotional processing, motor function [73,74], or the sensorimotor system, which can be tested by auditory brainstem response (ABR) [75,76]. Together, these findings indicate that increased locomotor activity, cognitive dysfunction, and abnormal anxiety in *Grin1*^Rgsc174^/*Grin1*^+^ mice may recapitulate some aspects of schizophrenia, ADHD, and bipolar disorder, while other aspects of these disorders, such as social withdrawal and deficits in sensorimotor gating [77,78], are not represented in *Grin1*^Rgsc174^/*Grin1*^+^ mice. Further studies would be important to clarify the behavioral abnormalities related to schizophrenia or ADHD in specific brain regions. In particular, in the hippocampus, the cognitive dysfunction could be assessed by electrophysiological analyses on CA pyramidal neurons or DG granule cells, biochemical analysis on signaling molecules that are involved in synaptic plasticity, and etc. [35,38]. In the basal ganglia, the hyperactivity can be analyzed by several methods, such as a histological analysis on dopaminergic neurons and quantification of the dopamine release using microdialysis technique [35,38,79].

In our previous studies, the mutant mouse strains, αCaMKII HKO, Shn-2 KO, and SNAP-25 KI mice, exhibited severe impairments in working memory and increased locomotor activity, which are abnormal behaviors related to schizophrenia, and displayed the “immature dentate gyrus (iDG)” phenotype, in which DG granule cells fail to mature [35-38]. In the present study, we assessed whether *Grin1*^Rgsc174^/*Grin1*^+^ mice demonstrate similar maturation abnormalities in DG granule cells. Mouse strains with iDG show a characteristic marker expression pattern, upregulation of *Drd1a*, and downregulation of *Dsp*, *Tdo2*, and *Calb1*[35-38]. Two out of four marker genes (downregulation of *Calb1* and *Tdo2*) in *Grin1*^Rgsc174^/*Grin1*^+^ mice were expressed consistently with this expression pattern, suggesting that *Grin1*^Rgsc174^/*Grin1*^+^ mice may partially have the iDG phenotype. There is also the possibility that the downregulation of the two genes is caused by the effect(s) of NMDA receptor mutation that are independent of the maturation abnormality of granule cells. Further studies, such as histological and physiological analyses, are needed to confirm the iDG phenotype of *Grin1*^Rgsc174^/*Grin1*^+^ mice.

## Conclusions

*Grin1*^Rgsc174^/*Grin1*^+^ mice exhibited behavioral abnormalities, including increased locomotor activity, abnormal anxiety-like behavior, a mild deficit in working memory, and severely impaired fear memory. They partially recapitulate the symptoms of ADHD, schizophrenia, and bipolar disorder. *Grin1*^Rgsc174^/*Grin1*^+^ mice show a unique profile of abnormal behaviors and may represent a subpopulation of patients with these psychiatric disorders.

## Methods

### Animals and experimental design

*Grin1*^Rgsc174^/*Grin1*^+^ mice, kindly provided by the RIKEN BioResource Center (Tsukuba, Japan), were backcrossed with C57BL/6J for six generations. Rgsc174 is an identification code for ENU mutant mouse strains established in the RIKEN Genome Science Center (RGSC; Yokohama, Japan). All behavioral tests were carried out with male mice that were at least 10 weeks old at the start of testing (Table 4). Wild-type littermates were used as controls for the experiments. The mice were group housed (2–4 mice per cage) in a room with a 12-hr light/dark cycle (lights on at 7:00 a.m.) with access to food and water *ad libitum*. The room temperature was kept at 23±2°C. Behavioral testing was performed between 9:00 a.m. and 6:00 p.m. After the tests, all apparatuses were cleaned with diluted sodium hypochlorite solution to prevent a bias due to olfactory cues. Experiments were conducted in the following sequence: light/dark transition, open field, hot plate, elevated plus maze, general health, the neurological screen, social interaction in open field, rotarod, Crawley’s sociability and preference for social novelty, acoustic startle response/prepulse inhibition, Porsolt forced swim, gait analysis, eight-arm radial maze, tail suspension, contextual and cued fear condition, and Social interaction in familiar environment. Each behavioral test was separated from the others by at least 1 day. All animal care, behavioral testing procedure, and animal experiments were approved by the Animal Research Committee, Graduate School of Medicine, Kyoto University (Permit No., MedKyo 09539) and the Institutional Animal Care and Use Committee of Fujita Health University (Permit No., I0741), based on the Law for the Humane Treatment and Management of Animals (2005) and the Standards Relating to the Care and Management of Laboratory Animals and Relief of Pain (2006). Every effort was made to minimize the number of animals used.

**Table 4:** **Comprehensive behavioral test battery of*Grin1***^**Rgsc174**^/***Grin1***^+^**mice**

**Test**	**Age (w)**	**Number of samples**	**Figure/Table**
Light–dark transition test	10	C, 11; M, 10	Figure 3
Open field test	10	C, 11; M, 10	Figure 2
Hot plate test	11	C, 11; M, 10	Figure 1
Elevated plus maze test	11	C, 11; M, 9	Figure 3
General health test	12	C, 11; M, 10	Figure 1
Social interaction test in novel environment	12	C, 5; M, 3*	Figure 4
Rotarod test	12	C, 11; M, 10	Figure 1
Crawley’s three-chamber social approach test	12-13	C, 11; M, 10	Figure 4
Acoustic startle response test	14	C, 11; M, 10	Figure 7
Prepulse inhibition test	14	C, 10; M, 7	Table 2
Porsolt forced swim test	15	C, 11; M, 10	Figure 5
Gait analysis	27-28	C, 10; M, 7	Figure 1
Eight-arm radial maze test	37-40	C, 10; M, 7	Figure 6
Contextual and cued fear conditioning Test	46	C, 11; M, 10	Figure 6
Tail suspension test	46	C, 11; M, 10	Table 2
Social interaction test in familiar environment	48-49	C, 5; M, 3*	Figure 4

C: Controls.

M: Mutants.

* Number of pairs.

### Neurological screen

The neurological screen was performed as previously described [35]. The righting, whisker touch, and ear twitch reflexes were evaluated. A number of physical features, including the presence of whiskers or bald hair patches, were also recorded.

### Neuromuscular strength

Neuromuscular strength was evaluated with the grip strength test and wire hang test. A grip strength meter (O'Hara & Co., Tokyo, Japan) was used to assess forelimb grip strength. Mice were lifted and held by their tail so that their forepaws could grasp a wire grid. The mice were then gently pulled backward by the tail with their posture parallel to the surface of the table until they released the grid. The peak force applied by the forelimbs of the mouse was recorded in Newtons (N). Each mouse was tested three times, and the greatest value measured was used for the statistical analysis. In the wire hang test, the mouse was placed on a wire mesh that was then inverted and waved gently, so that the mouse gripped the wire. Latency to fall was recorded, with a 60 sec cut-off time.

### Hot plate test

The hot plate test was used to evaluate sensitivity to a painful stimulus. Mice were placed on a 55.0 (± 0.3)°C hot plate (Columbus Instruments International, Columbus, OH), and latency to the first hind-paw response was recorded. The hind-paw response was defined as either a foot shake or a paw lick.

### Gait analysis (front and hind paws)

The gait of adult mice during spontaneous walk/trot locomotion was analyzed using the DigiGait™ Imaging System (Mouse Specifics Inc, Watertown, MA). In this system, mice walking on a motorized transparent treadmill belt are recorded on video, and the software automatically identifies the stance and swing components of the stride, and calculates stance width, stride length, step angle, and paw angle. Equivalent stride times for the fore and hind paws were composed of a shorter stance and a longer swing time. Peak vertical reaction force increased with decreasing stance time, and the results of the forelimbs were approximately 5% greater than those of the hind paws over the whole stance time range studied.

### Light/dark transition test

A light/dark transition test was conducted as previously described [80]. The apparatus used for the light/dark transition test was composed of a cage (21 × 42 × 25 cm) divided into two sections of equal size by a partition with a door (O'Hara & Co.). One chamber was brightly illuminated (390 lux), whereas the other chamber was dark (2 lux). Mice were placed into the dark side and allowed to move freely between the two chambers through an open door for 10 min. The total number of transitions, latency to first enter the light chamber, distance traveled, and time spent in each chamber were recorded by ImageLD software (see 'Data analysis').

### Elevated plus maze test

An elevated plus-maze test was conducted as previously described [81]. The elevated plus-maze consisted of two open arms (25 × 5 cm) and two enclosed arms of the same size with 15-cm high transparent walls. The arms and central square were made of white plastic plates and elevated 55 cm above the floor. To minimize the likelihood of animals falling from the apparatus, 3-mm-high Plexiglas walls surrounded the sides of the open arms. Arms of the same type were located opposite from each other. Each mouse was placed in the central square of the maze (5 × 5 cm), facing one of the closed arms. Mouse behavior was recorded during a 10-min test period. The number of entries into an arm and the time spent in the open and enclosed arms were recorded. The percentage of entries into open arms, the time spent in open arm (s), the number of total entries, and the total distance traveled (cm) were analyzed. Data acquisition and analysis were performed automatically using Image EP software (see 'Data analysis').

### Open field test

Locomotor activity was measured using an open field test. Each mouse was placed in the corner of the open field apparatus (40 × 40 × 30 cm; Accuscan Instruments, Columbus, OH). The chamber of the test was illuminated at 100 lux. The total distance traveled (in cm), vertical activity (rearing measured by counting the number of photobeam interruptions), and time spent in the center area (20 × 20 cm), and beam-break counts for stereotyped behaviors were recorded. If the animal broke the same beam (or set of beams) three times, then the monitor considers the animal to have exhibited stereotypic activity, including grooming and head bobbing. The stereotypy count is the number of beam breaks that occur during this period of stereotypic activity. The data were collected for 120 min.

### Social interaction test in a novel environment

In the social interaction test, two mice of identical genotypes that were previously housed in different cages were placed in a box together (40 × 40 × 30 cm) and allowed to explore freely for 10 min [82]. Because a pair of mice was used as a sample in the test, the number of samples is half. Social behavior was monitored with a CCD camera connected to a Macintosh computer. Analysis was performed automatically using ImageSI software (see 'Data analysis'). The total number of contacts, total duration of active contacts, total contact duration, mean duration per contact, and total distance traveled were measured. The active contact was defined as follows. Images were captured at 3 frames per second, and distance traveled between two successive frames was calculated for each mouse. If the two mice made contact and if the distance traveled by either mouse was longer than 4 cm, the behavior was considered as an “active contact”.

### Social interaction test in home cage

Social interaction monitoring in home cage was conducted as previously described [54]. The system comprised the home cage (29 × 18 × 12 cm) and a filtered cage top, separated by a 13-cm high metal stand containing an infrared video camera attached at the top of the stand. Two mice of the same genotype that had been housed separately were placed together in a home cage. Their social behavior was then monitored for 1 week. Output from the video camera was fed into a Macintosh computer. Images from each cage were captured at a rate of one frame per second. Social interaction was measured by counting the number of particles detected in each frame; two particles indicated that the mice were not in contact with each other, while one particle (i.e., the tracking software could not distinguish two separate bodies) indicated contact between the two mice. We also measured locomotor activity during these experiments by quantifying the number of pixels that changed between each pair of successive frames. Analysis was performed automatically using ImageHA software (see 'Data analysis').

### Crawley’s sociability and preference for social novelty test

The test for sociability and preference for social novelty is a well-designed method to investigate the complex genetics of social behaviors [83]. The apparatus comprised a rectangular, three-chambered box and a lid containing an infrared video camera (O'Hara & Co.). Each chamber was 20 × 40 × 22 cm and the dividing walls were made from clear Plexiglass, with small square openings (5 × 3 cm) allowing access into each chamber. An unfamiliar C57BL/6J male (stranger 1) that had no prior contact with the subject mouse was placed in one of the side chambers. The placement of stranger 1 in the left or right side chambers was systematically alternated between trials. The stranger mouse was enclosed in a small, circular wire cage, which allowed nose contact between the bars but prevented fighting. The cage was 11-cm high, with a bottom diameter of 9 cm and bars spaced at 0.5 cm intervals. The subject mouse was first placed in the middle chamber and allowed to explore the entire social test box for 10 min. The amount of time spent within a 5-cm distance of the wire cage in each chamber and the time spent in each chamber was measured with the aid of a camera fitted on top of the box. After the first 10 min, each mouse was tested in a second 10-min session to quantify social preference for a new stranger. A second, unfamiliar mouse was placed in the chamber that had been empty during the first 10-min session. This second stranger was enclosed in an identical small wire cage. The test mouse had a choice between the first, already-investigated unfamiliar mouse (stranger 1), and the novel unfamiliar mouse (stranger 2). As described above, the amount of time spent within a 5-cm distance of each wire cage and in each chamber during the second 10-min session was recorded. The stranger mice used in this experiment were 8- to 12-week-old C57BL/6J male mice that were not littermates. Analysis was performed automatically using ImageCSI software (see 'Data analysis').

### Contextual and cued fear conditioning test

Each mouse was placed in a test chamber (26 × 34 × 29 cm) inside a sound-attenuated chamber and allowed to explore freely for 2 min. A 60 dB white noise, which served as the conditioned stimulus (CS), was presented for 30 sec, followed by a mild footshock (2 sec, 0.5 mA) serving as the unconditioned stimulus (US). Two more CS-US pairings were presented with a 2-min inter-stimulus interval. Context testing was conducted 24 h after conditioning in the same chamber. Cued testing with altered context was performed after conditioning using a triangular box (35 × 35 × 40 cm) made of white opaque Plexiglas, which was located in a different room. The chamber of the test was illuminated at 100 lux. Data acquisition, control of stimuli (i.e., tones and shocks), and data analysis were performed automatically using ImageFZ software (see 'Data analysis'). Images were captured at 1 frame per second. For each pair of successive frames, the amount of area (pixels) that the mouse moved was measured. When this area was below a certain threshold (i.e., 20 pixels), the behavior was judged as ‘freezing.’ When the amount of area equaled or exceeded the threshold, the behavior was considered as ‘non-freezing.’ The optimal threshold (amount of pixels) to judge freezing was determined by adjusting it to the amount of freezing measured by human observation. ‘Freezing’ that lasted less than the defined time threshold (i.e., 2 sec) was not included in the analysis. The parameters were constant for all mice assessed.

### Eight-arm radial maze

The eight-arm radial maze test was performed using fully automated eight-arm radial maze apparatuses [35] (O'Hara & Co.). The floor of the maze was made of white plastic, and the wall (25-cm high) consisted of transparent plastic. Each arm (9 × 40 cm) radiated from an octagonal central starting platform (perimeter 12 × 8 cm) like the spokes of a wheel. Identical food wells (1.4-cm deep and 1.4-cm in diameter) with pellet sensors were placed at the distal end of each arm. The pellet sensors were able to automatically record the pellet intake of the mice. The maze was elevated 75 cm above the floor and placed in a dimly-lighted room with several extra-maze cues. During the experiment, the maze was maintained in a constant orientation. One week before pre-training, the animals were deprived of food until their body weight was reduced to 80% to 85% of their initial levels. In the pre-training, each mouse was placed in the central starting platform and allowed to explore and consume food pellets scattered over the whole maze for a 30-min period (one session per mouse). After completion of the initial pre-training, the mice received another pre-training to retrieve a food pellet from each food well after it had been placed at the distal end of each arm. A trial was finished when the mouse consumed the pellet. This procedure was repeated eight times, using eight different arms, for each mouse. After these pre-training trials, the actual maze acquisition trials were performed. In the spatial working memory task of the eight-arm radial maze, all eight arms were baited with food pellets. Mice were placed on the central platform and allowed to obtain all eight pellets within 25 min. A trial was terminated immediately after all eight pellets were consumed or 25 min had elapsed. An 'arm visit' was defined as travelling more than 5 cm from the central platform. The mice were confined at the central platform for 5 sec after each arm choice (without delay). The animals went through one trial per day (22 trials total). For each trial, arm choice, latency to obtain all pellets, distance traveled, number of different arms chosen within the first eight choices, the number of arm revisited, and omission errors were automatically recorded. The number of different arms chosen during the first eight choices is considered a measure of working memory, and to be relatively independent of locomotor activity levels [84-86]. To increase the difficulty of the task, in the 20th block of trials, a 30-sec delay was provided after four pellets had been taken by confining the mice in the center platform. During the 21st and 22nd blocks of the trial, the delay period was extended to 2 min and 5 min, respectively. A trial was terminated immediately after all of the pellets were consumed or 25 min had elapsed. After each trial, the maze was cleaned with water. The locations of the maze arms were randomly relocated after each session to prevent animals from using intra-maze cues. Data acquisition, control of guillotine doors, and data analysis were performed by ImageRM software (see 'Data analysis').

### Startle response prepulse inhibition tests

A startle reflex measurement system was used (O'Hara & Co.) to measure the startle response and prepulse inhibition. A test session began by placing a mouse in a plastic cylinder where it was left undisturbed for 10 min. White noise (40 msec) was used as the startle stimulus for all trial types. The startle response was recorded for 140 msec (measuring the response every 1 msec) starting from the onset of the prepulse stimulus. The background noise level in each chamber was 70 dB. The peak startle amplitude recorded during the 140-msec sampling window was used as the dependent variable. A test session consisted of six trial types (i.e., two types for startle stimulus only trials, and four types for prepulse inhibition trials). The intensity of the startle stimulus was 110 or 120 dB. The prepulse sound was presented 100 msec before the startle stimulus, and its intensity was 74 or 78 dB. Four combinations of prepulse and startle stimuli were used (74–110, 78–110, 74–120, and 78–120). Six blocks of the six trial types were presented in pseudorandom order such that each trial type was presented once within a block. The average inter-trial interval was 15 sec (range: 10–20 sec).

### Porsolt forced swim test

The Porsolt forced swim test apparatus consisted of four Plexiglass cylinders (20-cm high × 10-cm diameter). A nontransparent panel separated the cylinders to prevent the mice from seeing each other (O'Hara & Co.). The cylinders were filled with water (23°C) up to a height of 7.5 cm. Mice were placed into the cylinders, and the immobility and distance traveled were recorded over a 10-min test period. Images were captured at one frame per second. For each pair of successive frames, the amount of area (pixels) that the mouse moved in was measured. When the amount of area was below a certain threshold, mouse behavior was judged as “immobile.” When the amount of area equaled or exceeded the threshold, the mouse was considered as “moving.” The optimal threshold used for judging was determined through adjustments it to the amount of immobility measured by human observation. Immobility lasting for less than 2 sec was not included in the analysis. Retention tests were administered 24 h after training. Data acquisition and analysis were performed automatically using ImageTS software (see 'Data analysis').

### Rotarod test

Motor coordination and balance were tested with the rotarod test. The rotarod test, using an accelerating rotarod (UGO Basile North America Inc., Collegeville, PA), was performed by placing mice on rotating drums (3-cm diameter) and measuring the time each animal was able to maintain its balance on the rod. The speed of the rotarod accelerated from 4 to 40 rpm over a 5-min period. The animals went through three trials per day on two consecutive days. The trials were separated by more than 1-hr intertrial intervals.

### Quantitative RT-PCR

Quantitative RT-PCR analysis was conducted as previously described [35]. Total RNA was isolated from the hippocampi of 27- to 29-wk-old *Grin1*^Rgsc174^/*Grin1*^+^ mice and wild-type mice. First-strand cDNA was synthesized from 1 μg of DNase I-treated total RNA using the Superscript® VILO™ cDNA synthesis kit (Life Technologies, Grand Island, NY). The expression of related genes was quantified using SYBR GreenER qPCR SuperMix for ABI PRISM (Life Technologies) following the instructions of the manufacturer. Quantitative PCR was performed using ABI PRISM7700 (Life Technologies) with the following conditions: 2 min at 50°C and 10 min at 95°C, followed by 40 cycles of 15 sec at 94°C and 1 min at 60°C. β-actin was amplified from all samples to normalize expression. The following primer sequences were obtained from the Primer Bank [87-89] (http://pga.mgh.harvard.edu/primerbank/index.html): calbindin-28K (56–185); desmoplakin (7–113); tryptophan 2,3-dioxygenase (1–105); Drd1a (133–251); and β-actin (851–962). The Ct values used were the mean values of triplicates.

### Data analysis

The applications used for the behavioral studies (ImageLD, ImageSI, ImageTS, ImageCSI, ImageRM, ImageFZ) were developed by Dr. Tsuyoshi Miyakawa (available through O’Hara & Co.) based on the NIH Image program (NIH, Bethesda, MD, available at http://rsb.info.nih.gov/nih-image/) and ImageJ software (Imagejdev.Org, available at http://imagejdev.org/). Statistical analysis was conducted using StatView software (SAS Institute, Cary, NC). Data were analyzed by one-way analysis of variance (ANOVA), two-way repeated measures ANOVA, analysis of covariance (ANCOVA), the paired *t*-test, or Pearson’s correlation coefficient. Values in graphs are expressed as the mean ± SEM.

### Availability of supporting data

Dataset, such as experimental date, age, raw data, and summary data (mean ± SEM), of the behavioral tests are available in the mouse phenotype database repository, http://www.mouse-phenotype.org/.

## Competing interests

The authors declare that they have no competing interests.

## Authors’ contributions

TM was responsible for the original conception and overall design of the research, while T F, Y W, S W, and K Takao established the congenic mice. K Takao, K Toyama, and TM performed the behavioral analysis of the mice. JU conducted quantitative RT-PCR. JU, HK, and TM wrote the manuscript. All authors read and approved the final manuscript.
